# The common enteric bacterial pathogens and their antimicrobial susceptibility pattern among HIV-infected individuals attending the antiretroviral therapy clinic of Hawassa university hospital, southern Ethiopia

**DOI:** 10.1186/s13756-017-0288-7

**Published:** 2017-12-22

**Authors:** Ayele Kebede, Solomon Aragie, Techalew Shimelis

**Affiliations:** 1 Department of Biology, Hawassa University, College of Natural and Computational Sciences, P.O. Box – 1560, Hawassa, Ethiopia; 2 Department of Medical Laboratory Science, Hawassa University, College of Medicine and Health Sciences, P.O. Box– 1560, Hawassa, Ethiopia

**Keywords:** Enteric bacterial infection, HIV, Antimicrobial drug resistance, Ethiopia

## Abstract

**Background:**

The frequent occurrence of bacterial gastroenteritis among HIV-infected individuals together with increased antimicrobial drug resistance pose a significant public health challenge in developing countries. This study aimed to determine the prevalence of enteric bacterial pathogens and their antimicrobial susceptibility pattern among HIV-infected patients in a tertiary hospital in southern Ethiopia.

**Methods:**

A hospital-based cross-sectional study was conducted at Hawassa University Comprehensive Specialized Hospital from February to May, 2016. A consecutive 215 HIV-infected patients, with complaints of gastrointestinal tract disease, were enrolled. Data on socio-demography and related factors was collected using a structured questionnaire. A stool sample was collected from each study participant and cultured to isolate enteric bacterial pathogens; isolates were characterized using biochemical tests. Antimicrobial susceptibility was determined using the Kirby- Bauer disk diffusion technique.

**Results:**

Out of 215 patients, 27(12.6%) were culture positive for various bacterial pathogens. *Campylobacter* species was the most common bacterial isolate (6.04%), followed by *Salmonella* species (5.1%). The majority of isolates was sensitive to norfloxacin, nalidixic acid, gentamicin, ceftriaxone and ciprofloxacin and showed resistance to trimethoprim sulfamethoxazole (SXT) and chloramphenicol. Consumption of raw food was the only risk factor found to be significantly associated with enteric bacterial infection (crude odds ratio 3.41 95% CI 1.13–10.3).

**Conclusions:**

The observed rate of enteric bacterial pathogens and their antimicrobial resistance pattern to the commonly prescribed antibiotics highlights the need to strengthen intervention efforts and promote rational use of antimicrobials. In this regard, the need to strengthen antimicrobial stewardship efforts should be emphasized to slow grown antimicrobial resistance among this population group.

## Background

Human immunodeficiency virus (HIV) associated immunosuppression increases the vulnerability of patients to various infections [1]. Enteric bacterial pathogens such as *Salmonella* species, *Campylobacter* species, *Shigella* species, *Clostridium difficile* and different strains of *Escherichia coli* have been identified as etiologic agents with the potential to cause severe illness in HIV-infected patients [2, 3]. Symptoms, duration and potential for severe manifestations of enteric bacterial infections are influenced by many factors including immunity status (measured by CD4+ T cell count) and the use of highly active antiretroviral therapy (HAART) and prophylaxis. Previous research showed that patients with lower CD4+ T cell counts are more likely experience bacterial diarrhea [4]. Bacteremia due to *Salmonella*, *Campylobacter* and *Shigella* was also more common in those with lower CD4+ T cell counts [5, 6].

Gastrointestinal tract (GIT) illnesses such as diarrhea affect up to 95% of persons with AIDS, frequently causing malabsorption, significant weight loss, higher rates of extra-intestinal infections, and increased mortality in developing countries [7]. The introduction of HAART and prophylactic management of opportunistic infections has brought substantial improvements in health and life expectancy of PLHIV. Several reports from developed countries have shown the decline in diarrheal episodes (specifically bacterial diarrhea) [4, 6]. However, these protective roles of HAART were not demonstrated in some other studies [3, 8].

An increasing rate of bacterial resistance to drugs commonly used to treat enteric infections further complicates the management of disease in PLWHIV. Due to the frequent exposure to antibiotic therapy and prophylaxis, HIV-infected patients might carry resistant bacterial strains [9]. In some studies, enteric bacterial pathogens with higher rate of resistance to antimicrobials were isolated from PLWHIV [10, 11].

In Ethiopia, information about the epidemiology of enteric bacterial pathogens in PLHIV is scant. A study that investigated HIV-infected patients in the southwest part of the country (Jimma) during the pre-HAART period showed bacterial pathogens such as *Salmonella*, *Shigella*, *Campylobacter* and *E. coli* were common [12]. In the same study, isolates were found to be less sensitive to tetracycline, chloramphenicol, gentamicin, and nalidixic acid. The present study aimed to identify bacterial pathogens and their antibiotic susceptibility pattern among HIV-infected patients attending a largest hospital in southern Ethiopia. The findings of this study will help generate evidence to inform antimicrobial stewardship efforts, and provide location –specific comparator information for similar studies undertaken in other populations.

## Methods

### Study design and setting

A hospital-based cross-sectional study was conducted at Hawassa University Comprehensive Specialized Hospital (HUCSH) from February to May, 2016. The hospital is situated at Hawassa, the capital city of the Southern Nation, Nationalities and Peoples’ Region. The hospital is the largest in the administrative region with bed capacity of 400. The antiretroviral therapy (ART) clinic in the facility offers treatment service for new and follow-up HIV-infected clients. Clinical and immunological assessments (CD4+ T cell count) at enrolment and at three-monthly intervals help determine patients’ eligibility for HAART. HIV-infected patients with clinical indications of GIT diseases are routinely investigated for intestinal parasites in the hospital. Stool bacterial culturing is not performed on routine basis.

### Population

The study population consisted of HIV-infected patients attending the ART clinic at HUCSH during the study period and presenting with signs and symptoms of GIT disease. Patients aged <18 years, or who could not provide a stool sample or who had taken antimicrobial treatment (except trimethoprim-sulphamethoxazole (SXT) prophylaxis) within two weeks prior to the time of data collection were excluded.

### Sample size and sampling technique

The sample size was estimated using a single proportion formula, and assuming a prevalence of enteric bacterial infection in HIV- infected patients of 16% [12], 95% level of confidence and 5% margin of error. A convenient sampling technique was used to enroll study participants, in which consecutive HIV-infected patients eligible for enrollment were invited to participate.

### Data collection

#### Socio- demography and clinical data

A structured questionnaire was used to collect socio-demographical (age, sex, residence, educational status) and other related factors (hand washing practice, habit of consuming raw food, own domestic animals, availability and usage of latrine, source and treatment of water for drinking). Patients were also asked for complaints of diarrhea (passing three or more loose or liquid stools over a 24-h period). Data on recent level of CD4+ T cell count, HAART status and prophylactic usage of SXT was obtained from patients’ medical records.

#### Laboratory testing

##### Bacterial isolation and characterization

A single stool sample was collected from each study participant using a sterile and disinfectant-free container. Stool samples were cultured on MacConkey (MAC) and Xylose Lysine Deoxychocolate (XLD) agar media after enrichment on Selenite-F Broth (Oxoid, United Kingdom). Further, blood free *Campylobacter* selective agar with Cefoperazone, Amphotericin B and Teicoplanin (CAT) selective supplement (Himedia, Ltd) was inoculated for isolation of *Campylobacter* species XLD and MAC agar plates were incubated aerobically at 37 °C for 24 h. Agar plates for isolation of *Campylobacter* species were incubated under microaerophilic conditions (5–10% O_2_ and 10% CO_2_ concentration) at 37 °C for 48 h. To create microaerophilic condition, the inoculated agar plates were kept in a gas jar containing gas generating kit (Campy Gen™, Oxoid, United Kingdom). Isolates were characterized using biochemical tests. Serotyping was performed for *Salmonella, Shigella* and *E. coli* using commercial anti-sera.

##### Antimicrobial susceptibility test

Antibiotic susceptibility testing was performed using the Kirby-Bauer disc diffusion method according to the recommendation of the Clinical and Laboratory Standards Institute (CLSI) (CLSI, 2011). In brief, 1–3 similar bacterial colonies were inoculated into a nutrient broth to prepare inoculums; broths were incubated at 37 °C for 4 h. Turbidity of broths was standardized at 0.5 McFarland using sterile phosphate buffered saline (pH, 7.2). The standardized suspension was inoculated on Mueller-Hinton agar plates. Defibrinated sheep blood (5%) was added for susceptibility testing of *Campylobacter* isolates. Antibiotic discs representing commonly prescribed antimicrobials in the study area were tested; these included chloramphenicol (30 μg), ciprofloxacin (5 μg), trimethoprim-sulfamethoxazole (1.25/23.75 μg), erythromycin (15 μg), gentamicin (10 μg), nalidixic acid (30 μg), norfloxacin (30 μg), tetracycline (30 μg), and ceftriaxone (30 μg)*.* Plates were incubated aerobically at 37 °C for 16–18 h; the incubation for *Campylobacter* species was done under microaerophilic condition for 48 h. The diameter of inhibition zone was measured using a caliper and interpreted according to the standard (CLSI, 2011). A reference strain of *E. coli* (ATCC-25922) was tested as a control.

### Statistical analysis

Data entry and analysis were performed using SPSS Version 20 software. Results were summarized using percentages and frequencies. Binary logistic regression analyses were performed and crude odds ratio with 95% confidence interval (CI) were calculated to measure the strength of association between the dependent and independent variables.

## Results

### Participants’ socio-demographic and clinical profile

A total of 215 HIV-infected individuals were enrolled in this study. The participants’ mean age was 30.3 years (range, 18–55 years). The most common age group represented was those aged 45 to 54 years (34.8%). Of the study participants, 52% were females and 68.8% were residents of urban areas (Table 1). HIV infected individuals with a CD4+ T cell count ≤350 cells/mm^3^ accounted for 41.7%. Within this group, 91.8% were taking SXT prophylaxis. The remaining individuals had either discontinued or had not started taking the prophylaxis. The majority of the respondents (66%) received HAART with the mean duration of treatment having being 39 months (range 2–84 months). Participants who reported having diarrhea accounted for 47.4% (Table 2).

**Table 1:** The occurrence and distribution of pathogenic enteric bacteria by socio-demographic characteristics in HIV- infected individuals at HUCSH, 2016

Characteristics	Number (%) tested (*n* = 215)	Number (%) positive for enteric bacteria (*n* = 27)	Crude odds ratio (95% CI)
Sex			
Male	103 (47.9)	11 (10.7)	0.75 (0.32–1.77)
Female	112 (52.1)	16 (14.2)	1
Age			
18–24	25 (11.6)	4 (16.0)	1
25–34	50 (23.3)	4 (8.0)	0.50 (0.11–2.17)
35–44	58 (27.0)	10 (17.2)	1.08 (0.31–3.76)
45–54	70 (32.6)	8 (11.4)	0.71 (0.20–2.58)
≥ 55	9 (4.2)	1 (11.1)	0.07 (0.01–0.65)
Residence			
Urban	148 (68.8)	17 (11.4)	1
Rural	67 (31.2)	10 (15)	0.69 (0.28–1.66)
Educational Status			
Illiterate	49 (22.8)	5 (10)	1
Primary	108 (50.2)	12 (11.1)	0.92 (0.31–2.75)
Secondary and above	57 (27.0)	10 (17.2)	1.08 (0.31–3.76)

**Table 2:** The distribution of enteric bacterial pathogens in relation to CD4+ T cell count, diarrhea status and treatment status in HIV- infected individuals at HUCSH, 2016

Characteristics	Number (%) tested (n = 215)	Number (%) positive for enteric bacteria (n = 27)	Crude odds ratio (95% CI)
CD4+ T cell count			
≤ 350 cells/mm^3^	88 (40.9)	12 (13.6)	1.18 (0.52–2.66)
> 350 cells/mm^3^	127 (59.1)	15 (11.8)	1
Diarrhea status			
Diarrhea	102 (47.4)	21 (20.5)	4.62 (1.78–11.98)^a^
No-diarrhea	113 (52.6)	6 (5.3)	1
ART status			
Not taking HAART	82 (38.1)	11 (13.4)	0.99 (0.44–2.22)
Taking HAART	133 (61.9)	18 (13.5)	1
SXT prophylaxis			
Not-taking	135 (62.8)	18 (13.3)	1.21 (0.52–2.85)
Taking	80 (37.2)	9 (11.3)	1

^a^statistically significant

SXT, cotrimoxazole; HAART, highly active anti-retroviral therapy; CD, cluster of differentiation; CI, confidence interval

### Bacterial profile and distribution

Laboratory analysis of the stool samples showed that 12.6% of the study participants had at least one enteric bacterial pathogen. The most frequent isolates were *Campylobacter* species (6.04%), followed by *Salmonella* species (5.1%). The rates of *Shigella* species and enterohaemorrhagic *E. coli* (EHEC) were 1.3% and 0.9%, respectively. The majority of isolates (80%) was detected among participants who reported having diarrhea at the time of data collection (Table 3).

**Table 3:** Enteric bacterial pathogens isolated from HIV-infected individuals with or without diarrhea in HUCSH, 2016

Isolates	Diarrhea status		Total (%) (n = 215)
	Diarrhea (%) (*n* = 102)	No-diarrhea (%) (*n* = 113)	
*Salmonella* species	7 (6.9)	4 (3.5)	11 (5.1)
*S. Typhi*	3	0	3
*S. Paratyphi A*	2	2	4
*S. Typhimurium*	2	2	4
*Shigella* species	3 (2.9)	0	3 (1.4)
*S. flexneri*	2	0	2
*S. dysenteriae*	1	0	1
*E. coli* (only EHEC strains*)*	2 (2)	0	2 (0.9)
O26:H11	1	0	1
O48:H21	1	0	1
*Campylobacter* species	11(10.8)	2 (1.8)	13 (6.04)
Total	23 (22.5)	6 (5.3)	29 (13.4)

EHEC, enterohaemorrhagic *Escherichia coli*

As presented in Tables 1 and 2, the association between diarrhea status and rate of enteric bacterial isolate was found to be statistically significant, which participants with diarrhea had a higher proportion of enteric bacterial pathogens than participants without diarrhea (crude odds ratio (COR) 4.62; 95% CI 1.78–11.98).

### The associated risk factors with enteric bacterial infections

The majority of the study participants used protected water sources for drinking (88.2%), owned a private latrine (95.8%), and consuming raw food (65.6%). The bacterial isolation rates were higher among participants, who sometimes used soap to wash hands at key hand washing times (15.4%), consumed raw food (16.3%), owned domestic animal at home (15.8%), and had no private latrine (22.2%). Only consumption of raw food was found to significantly increase the risk of having enteric bacterial infections among HIV-infected individuals (COR 3.41; 95% CI 1.13–10.3) (Table 4).

**Table 4:** The occurrence of enteric bacterial pathogens in relation to risk factors among HIV- infected individuals at HUCSH, 2016

Characteristics	Number (%) tested (n = 215)	Number (%) positive for enteric bacteria (n = 27)	Crude odds ratio (95% CI)
Hand washing practice			
Always	98 (45.6)	9 (9.2)	1
Sometimes	117 (54.4)	18 (15.4)	1.87 (0.76–4.59)
Consume raw food			
Yes	141 (65.6)	23 (16.3)	3.41 (1.13–10.3)^a^
No	74 (34.4)	4 (5.4)	1
Domestic animal at home			
Yes	114 (53.0)	18 (15.8)	1.92 (0.82–4.4)
No	101 (47.0)	9 (8.9)	1
Source of water for drinking			
Protected	191 (88.8)	22 (11.5)	1
Un-Protected	24 (11.2)	5 (20.8)	2.02 (0.69–5.96)
Use of treated drinking water			
Yes	92 (42.8)	9 (9.8)	1
No	123 (57.2)	18 (14.6)	1.77 (0.76–4.13)
Availability of private latrine			
Yes	206 (95.8)	25 (12.1)	1
No	9 (4.2)	2 (22.2)	2.07 (0.41–10.52)

^a^statistically significant

### Antimicrobial susceptibility pattern

It was observed that 8 of 13 tested *Campylobacter* isolates were found to be resistant to trimethoprim-sulfamethoxazol. Moreover, 8 of 11 *Salmonella* isolates were resistant to trimethoprim-sulfamethoxazol and chloramphenicol. The majority of isolates was sensitive to norfloxacin, nalidixic acid, gentamicin, ceftriaxone and ciprofloxacin (Table 5)*.*

**Table 5:** Antimicrobial resistance pattern of enteric bacterial isolates (*n* = 29) detected in stool samples of HIV-infected individuals in HUCSH, 2016

Antibiotics	*Campylobacter* spp. (*n* = 13)	*Salmonella* spp*.* (n = 11)	*Shigella* spp. (*n* = 3)	*E. coli (EHEC)* (n = 2)
Nalidixic acid	1	1	0	1
Gentamicin	0	1	1	0
Ceftriaxone	0	1	0	1
Tetracycline	3	7	3	1
Norfloxacin	0	0	0	1
Ciprofloxacin	1	1	0	0
Trimethoprim Sulfamethoxazol	8	8	2	2
Chloramphenicol	7	8	3	1
Erythromycin	3	6	2	1

## Discussion

A compromised immunity resulting from HIV infection hampers the intestinal defense against microbes. Consequently, enteric bacteria are common and more likely to cause diarrhea and other invasive conditions in HIV-infected patients than healthy individuals [13]. The rate of enteric bacterial isolates in the present study was shown to be 12.6%. A similar rate (16%) was reported in a previous study in southwest Ethiopia (Jimma) [12], and other settings outside Africa, including Cambodia (12.5%) [14] and Peru (14.5%) [2]. Rates reported in Uganda (19.2%) [15] and India (29%) [16] were higher than our findings. The rate of bacterial isolation among patients with diarrhea (22.5%) in the current study was comparable to findings in Peru (22%) [2], Thailand (17.4%) [17], and Uganda (21%) [15] but lower than rates reported from India (44%) [16] and South Africa (43.3%) [18]. The observed differences in rates of bacterial isolation could be attributed to differences in risk factors in various populations. The number of patients with diarrhea included in different studies could also affect the rate of enteric bacterial isolates as it was shown in the present study that majority of the pathogens (80%) were isolated from those patients who reported having diarrhea.

The predominance of *Campylobacter* species in the present study was similarly reported in South Africa [18] and England [3] although this bacterium was not isolated in other studies that employed similar laboratory technique [14, 16]. The rates of *Campylobacter* species in Uganda (3.5%) [15], in Peru (1.7%) [2], and in Brazil (1%) [19] were shown to be lower than our observation. The rate of *Salmonella* infection (5.1%) in the present study was higher than a finding in Peru (1%) [2], but lower than a result from Uganda (8.1%) [15]. The rate of *Shigella* species (1.3%) was also comparable with findings in India (1%) [16] and Cambodia (1.3%) [14], but lower than a result in Uganda (9.5%) [15]. The predominance of *S. flexineri* was consistent with results in Thailand and USA [4, 17]. Similar to the studies reported elsewhere [17, 20], only EHEC strains (O26:H11 and O48:H21) was identified in the present study even if reports [19, 21] on other strains of *E. coli* were also documented*.*

The occurrence of enteric bacterial infection in HIV-infected individuals is thought to be the net effect of socio-demographic as well as clinical status of the infected patients. Factors like hand washing practice, contact with pet animals, consumption of raw food and others have been indicated as potential factors for acquiring enteric bacterial infection [22]. In the current study, only a habit of consuming raw food was found to increase the odds of having enteric bacterial pathogens.

It was previously reported that a higher rate (80%) of enteric bacterial isolates among patients with CD4+ T cell count <200 cells**/**mm^3^ [4]. In contrast, the current study showed no significant difference in the rate of bacterial isolates among patients with CD4+ T cell count above and below 350 cells**/**mm^3^. Similar finding was reported in a study from England where 65.6% of the isolates were detected among patients with CD4+ T cell count >350 cells**/**mm^3^ [3].

The role of HAART in protecting against enteric infection through inhibition of viral replication and restoring immunity has been well documented [23]. However, the absence of association between the rate of isolation and HAART status in this study may be due to the frequent exposure of people living in poor hygienic condition to enteric bacterial infection that possibly diminishes the protective role of HAART in the present study participants. The limited effect of HAART in restoring GIT immunity was also reported [24].

Globally, increased bacterial resistance to antimicrobial agents is raising a significant concern for public health. The study showed that most bacterial isolates were resistant to tetracycline, SXT and chloramphenicol. Resistance is likely to have developed due to the unrestricted, frequent and inappropriate usage of antimicrobials in the study area. These drugs are commonly used for empirical treatment (SXT and chloramphenicol), as prophylactic before and after surgery (chloramphenicol together with other agents), and patients’ own self-medication (SXT and tetracycline) [25]. The present study showed that most of the isolates were sensitive to nalidixic acid, gentamicin, norfloxacin, ceftriaxone and ciprofloxacin.

Our study faces several limitations. First, as for any hospital-based study, our sample may have lacked representativeness and results may not be generalizable to all HIV-infected population in the study area. Second, the small sample size of the study likely limits its power.

## Conclusion

This study showed that enteric bacterial pathogens were common among HIV-infected individuals, particularly in those who experienced diarrhea. *Campylobacter* and *Salmonella* species were the predominant isolates. The observed significant association between consumption of raw food and enteric bacteria pathogens may hint at the importance of ingesting adequately cooked food to prevent infection. Most isolates were sensitive to nalidixic acid, gentamicin, norfloxacin, ceftriaxone and ciprofloxacin and a high proportion were resistant to SXT and chloramphenicol. The need to strengthen antimicrobial stewardship efforts should be emphasized to slow grown antimicrobial resistance among this population group.

## Abbreviations

AIDS: Acquired immunodeficiency syndrome
ART: Antiretroviral therapy
CLSI: Clinical and Laboratory Standards Institutes
EHEC: Enterohaemorrhagic *Escherichia coli*
GIT: Gastrointestinal tract
HAART: Highly active antiretroviral therapy
HIV: Human immunodeficiency virus
HUCSH: Hawassa University Comprehensive Specialized Hospital
MAC: MacConkey
MDR: Multidrug resistance
NTS: Non- typhoidal *Salmonella*
PLWHIV: People living with HIV
SXT: Trimethoprim-sulfamethoxazole
XLD: Xylose Lysine Deoxychocolate
